# COVID-19 vaccine uptake in US adults with respiratory diseases: A cross-sectional study

**DOI:** 10.1016/j.pmedr.2026.103430

**Published:** 2026-02-26

**Authors:** Moosa Tatar, Shahryar Karimi, Masoud Valizadeh, Abhishek Deshpande

**Affiliations:** a Department of Pharmaceutical Health Outcomes and Policy, University of Houston, Houston, TX, USA; b Department of Mathematical and Statistical Sciences, Marquette University, Milwaukee, WI, USA; c Options Clearing Corporation, Chicago, IL, USA; d Alice L. Walton School of Medicine, Bentonville, AR, USA

**Keywords:** COVID-19, Vaccine hesitancy, Respiratory disease, Influenza, Infectious disease, Immunization

## Abstract

**Objectives:**

Despite their proven effectiveness, COVID-19 vaccination rates remain low, particularly among individuals with respiratory diseases. We aimed to examine factors associated with vaccine uptake, focusing on respiratory conditions and prior respiratory vaccination history.

**Methods:**

We conducted a cross-sectional analysis using 2022 data from the Medical Expenditure Panel Survey. We used logistic regression to examine the association between COVID-19 vaccine uptake and various socioeconomic factors, respiratory conditions, and prior influenza vaccination in US adults.

**Results:**

Of 18,155 participants, 3083 (16.98%) were unvaccinated and 15,072 (83.02%) vaccinated. Unvaccinated individuals were more likely younger, male, non-Hispanic Black, immigrants, lower-income, less educated, Southern residents, and smokers. Prior influenza (OR = 1.82, 95% CI [1.39, 2.42]) and pneumonia vaccination (OR = 2.55, 95% CI [2.14, 3.04]) were linked to higher COVID-19 vaccination. Among those with respiratory disease, prior influenza (OR = 1.78, 95% CI [1.05, 3.21]) and pneumonia vaccination (OR = 2.09, 95% CI [1.50, 2.94]) predicted greater uptake. The highest rates were in individuals vaccinated for both (OR = 4.79, 95% CI [2.51, 9.37]). Respiratory diseases themselves were not significant predictors.

**Conclusions:**

Prior vaccination history and socioeconomic status are stronger predictors of COVID-19 vaccine uptake than respiratory disease history. Enhancing routine immunization and reducing socioeconomic disparities may improve coverage better than disease-specific approaches.

## Highlights

• • Prior vaccination predicts future COVID-19 vaccination uptake.
• • Socioeconomic factors influence COVID-19 vaccination uptake.
• • Respiratory disease does not predict COVID-19 vaccination uptake.

## 1 Introduction

Despite the widespread availability and demonstrated effectiveness of COVID-19 vaccines in preventing severe outcomes, many individuals continue to delay or refuse vaccination, and vaccination coverage remains suboptimal (Dudley et al., 2020; Islam et al., 2021; Lazarus et al., 2023; Loomba et al., 2021; *Medical Expenditure Panel Survey (MEPS*), 2024). Vaccine hesitancy persists as a significant public health challenge, particularly among minority populations and those with high social vulnerability (Khan et al., 2025; Tatar et al., 2023), mainly due to concern about vaccine safety and potential side effects (Khan et al., 2025; Aw et al., 2021; Chirico et al., 2022; Soares et al., 2021; Troiano and Nardi, 2021), and also barriers to vaccination access (Abba-Aji et al., 2022; Newman et al., 2025). Patients with prior respiratory diseases are at higher risk of developing severe COVID-19 and have a higher mortality rate compared to patients without a history of respiratory illness (Beltramo et al., 2021)., (Bouloukaki et al., 2024; Liang and Sun, 2023) Despite the assumption that the most vulnerable individuals (i.e., those with cancer, autoimmune diseases, and chronic lung diseases) would automatically accept the COVID-19 vaccination, individuals with serious comorbid conditions remain hesitant about the COVID-19 vaccine (Tsai et al., 2022).

Although the CDC recommends vaccination for high-risk individuals, including those with asthma, lung disease, pulmonary disease, cystic fibrosis, tuberculosis, smoking history, and transplant-related immunosuppression, vaccination uptake patterns among adults with respiratory conditions remain incompletely characterized (Centers for Disease Control and Prevention, 2025). Understanding factors that influence vaccination decisions in clinically and socially vulnerable populations is important for targeted public health interventions. Previous studies have examined COVID-19 vaccine hesitancy primarily in general populations or focused on single respiratory conditions (Chang et al., 2023; Tavakol et al., 2024). The relationship between prior vaccination (influenza, pneumonia) behaviors and COVID-19 vaccine acceptance across respiratory disease populations has been previously reported (Barri et al., 2025; Saiphoklang and Phadungwatthanachai, 2022; Tang et al., 2024). It also remains unclear how socioeconomic factors interact with respiratory disease status to influence vaccination decisions.

We hypothesized that prior respiratory vaccination history would be associated with increased COVID-19 vaccine uptake, and that this relationship might vary by respiratory disease status. Using nationally representative data could inform targeted vaccination strategies. We analyzed data from the 2022 Medical Expenditure Panel Survey (MEPS) to determine factors associated with COVID-19 vaccine uptake among US adults, with particular emphasis on respiratory diseases (i.e., asthma, bronchitis, or emphysema) and prior vaccination history (i.e., influenza and pneumonia) (*Medical Expenditure Panel Survey (MEPS*), 2024).

## 2 Methods

### 2.1 Study design and population

This retrospective cross-sectional study analyzed data from the MEPS for 2022 (*Medical Expenditure Panel Survey (MEPS*), 2024), the most recent year with publicly available data. MEPS is a nationally representative survey conducted by the Agency for Healthcare Research and Quality (AHRQ) that collects data on healthcare utilization, expenditures, insurance coverage, and demographic characteristics from households, medical providers, and employers across the United States (Beltramo et al., 2021; *Medical expenditure panel survey (MEPS). Medical Expenditure Panel Survey*, 2025).

The study population comprised individuals aged 17 and older with complete data on COVID-19 vaccination status and relevant covariates. The original dataset included 22,431 observations. There were 1014 observations with missing data (< 5%). Participants with missing data on key variables were excluded from the analysis. We analyzed individuals aged 17–85 years, consisting of 18,155 individuals. Unweighted analyses were performed to prioritize interpretability and analytical precision in examining associations between covariates and vaccination status, rather than generating nationally representative estimates. This approach is consistent with analytical frameworks focused on identifying predictors of health behaviors rather than population-level estimates. We conducted unweighted models to prioritize interpretability and analytical precision.

This study utilized publicly available, de-identified secondary data and did not involve direct contact with human participants. Therefore, it was exempt from institutional review board oversight in accordance with federal regulations governing human subjects research (45 CFR 46.104).

### 2.2 Measures

The primary outcome was COVID-19 vaccine uptake, defined as a binary variable indicating receipt of at least one dose of any COVID-19 vaccine versus no vaccination. This variable indicated whether individuals ever had a COVID-19 vaccine or booster shot. The primary exposure variable was history of respiratory disease, defined as the presence of at least one of the following conditions: asthma (asthma diagnosis), bronchitis (a person has had chronic bronchitis in the last 12 months), or emphysema (emphysema diagnosis). Individual binary variables for each respiratory condition and lung cancer were also examined. Respiratory diseases were identified using International Classification of Diseases, 10th Revision (ICD-10) diagnostic codes (asthma: J45; bronchitis: J20; emphysema: J43; lung cancer: C34) as recorded in MEPS administrative data and validated through medical provider reports. Please see Table A1 in the Appendix.

Additional binary indicators were constructed for history of influenza and pneumonia vaccination based on MEPS variable definitions, defined as having ever received an influenza or pneumonia vaccination, respectively. All outcome and covariate variables were derived from MEPS documentation and represent either administrative records or validated self-reported physician diagnoses, as specified in the survey methodology.

Covariates identified a priori as potential confounders based on existing literature included sociodemographic characteristics: age (continuous), sex assigned at birth, race/ethnicity, immigration status, household income level, educational attainment, geographic region, and smoking status. Complete variable definitions and coding specifications are provided in the supplementary materials (Table A1, Table A2).

### 2.3 Statistical analysis

Descriptive statistics were used to characterize the study population. Categorical variables are presented as frequencies and percentages, while continuous variables are presented as means and standard deviations. Differences between groups were assessed using chi-square tests for categorical variables and *t*-tests or ANOVA for continuous variables, as appropriate. To investigate associations between prior influenza vaccination, history of respiratory disease, and COVID-19 vaccine uptake, we conducted a series of multivariable logistic regression analyses.

First, we performed the primary analysis that included all participants and examined associations between COVID-19 vaccine uptake (dependent variable) and independent variables including, influenza vaccination history, pneumonia vaccination history, respiratory diseases (asthma, bronchitis, emphysema) and lung cancer while adjusting for relevant covariates (age, sex, race/ethnicity, immigration status, income, education, geographic region, and smoking status).

Second, we performed a subgroup analysis that included 1) individuals with at least one respiratory disease 2) individuals without any respiratory diseases, to examine the association between COVID-19 vaccine uptake and influenza vaccination history, pneumonia vaccination history, and lung cancer while adjusting for relevant covariates.

Third, we performed a subgroup analysis that included 1) individuals with prior influenza vaccination history 2) individuals without influenza vaccination history, to examine the association between COVID-19 vaccine uptake and respiratory disease, pneumonia vaccination, and lung cancer, and adjusting for relevant covariates (age, sex, race/ethnicity, etc.). In all multivariable logistic regression models, we estimated odds ratios (ORs) and 95% confidence intervals (CIs). Multicollinearity was assessed using variance inflation factors (VIFs), with values >5 indicating significant multicollinearity. Model fit was assessed using measures such as the pseudo-R-squared (Nagelkerke R2), Akaike Information Criterion (AIC), and Bayesian Information Criterion (BIC). Model performance was further evaluated using Monte Carlo cross-validation. The dataset was randomly split into training (70%) and testing (30%) subsets across 10 independent iterations. In each iteration, the model was trained on the training set and evaluated on the test set. The average prediction accuracy across all 10 iterations was used to estimate accuracy and out-of-sample performance.

All statistical analyses were performed using RStudio version 4.0.2 (R Core Team, 2020). A *p*-value of <0.05 was considered statistically significant. Given the secondary nature of this analysis using existing survey data, no formal sample size calculation was performed.

## 3 Results

Among 18,155 participants, 3083 (16.98%) were unvaccinated and 15,072 (83.02%) were vaccinated against COVID-19 (Table 1). Vaccination status was significantly associated with multiple demographic and health-related variables (*P* < 0.01). Unvaccinated individuals were more likely to be younger, with 18.42% aged 17–25 years and 18.94% aged 26–34 years, compared with 9.42% and 11.61%, respectively, among vaccinated individuals. Similar patterns were observed in the 35–54 age group (34.19% unvaccinated vs. 29.06% vaccinated). Conversely, older age groups were underrepresented among unvaccinated individuals: 14.63% aged 55–64 years and 13.82% aged ≥65 years, compared with 17.85% and 32.06%, respectively, among vaccinated individuals.

**Table 1:** Baseline characteristics by COVID-19 vaccination status in US adults (Medical Expenditure Panel Survey, 2022).

Variables	Not vaccinated	Vaccinated	Total	P-Value
Participant No. (%)	3083 (16.98%)	15,072 (83.02%)	18,155	
Age Group				< 0.01
17–25 years, No. (%)	568 (18.42%)	1420 (9.42%)	1988	
26–34 years, No. (%)	584 (18.94%)	1750 (11.61%)	2334	
35–54 years, No. (%)	1054 (34.19%)	4380 (29.06%)	5434	
55–64 years, No. (%)	451 (14.63%)	2690 (17.85%)	3141	
≥ 65 years, No. (%)	426 (13.82%)	4832 (32.06%)	5258	
Sex				< 0.01
Male, No. (%)	1535 (49.79%)	6856 (45.49%)	8391	
Female, No. (%)	1548 (50.21%)	8216 (54.51%)	9764	
Race/Ethnicity				< 0.01
White, non-Hispanic, No. (%)	1757 (56.99%)	8601 (57.07%)	10,358	
Black, non-Hispanic, No. (%)	523 (16.96%)	2101 (13.94%)	2624	
Hispanic or Latino, No. (%)	612 (19.85%)	3002 (19.92%)	3614	
Other/Multi-race, No. (%)	191 (6.20%)	1368 (9.08%)	1559	
Immigrant (foreign-born), No. (%)				< 0.01
No, No. (%)	2669 (86.57%)	11,904 (78.98%)	14,573	
Yes, No. (%)	414 (13.43%)	3168 (21.02%)	3582	
Income Level				< 0.01
Lower class: ≤ $30,000, No. (%)	1901 (61.66%)	6760 (44.85%)	8661	
Lower middle class: $30,001–$58,020, No. (%)	695 (22.54%)	3634 (24.11%)	4329	
Middle class: $58,021–$94,000, No. (%)	314 (10.18%)	2341 (15.53%)	2655	
Upper middle class: $94,001–$153,000, No. (%)	132 (4.28%)	1651 (10.95%)	1783	
Upper class: > $153,000, No. (%)	41 (1.33%)	686 (4.55%)	727	
Education Level				< 0.01
No degree, No. (%)	725 (23.52%)	2126 (14.11%)	2851	
Grade school, No. (%)	196 (6.36%)	493 (3.27%)	689	
High school, No. (%)	1462 (47.42%)	5318 (35.28%)	6780	
Some college, No. (%)	303 (9.83%)	1461 (9.69%)	1764	
Bachelor's degree, No. (%)	380 (12.33%)	5167 (34.28%)	5547	
Advanced degrees, No. (%)	17 (0.55%)	507 (3.36%)	524	
Region				< 0.01
Midwest, No. (%)	691 (22.41%)	2965 (19.67%)	3656	
Northeast, No. (%)	318 (10.31%)	2542 (16.87%)	2860	
South, No. (%)	1428 (46.32%)	5584 (37.05%)	7012	
West, No. (%)	646 (20.95%)	3981 (26.41%)	4627	
Respiratory Diseases (Asthma, Bronchitis, Emphysema)				0.35
No, No. (%)	2562 (83.10%)	12,630 (83.80%)	15,192	
Yes, No. (%)	521 (16.90%)	2442 (16.20%)	2963	
Asthma				0.48
No, No. (%)	2614 (84.79%)	12,857 (85.30%)	15,471	
Yes, No. (%)	469 (15.21%)	2215 (14.70%)	2684	
Bronchitis				0.55
No, No. (%)	3028 (98.22%)	14,829 (98.39%)	17,857	
Yes, No. (%)	55 (1.78%)	243 (1.61%)	298	
Emphysema				0.36
No, No. (%)	3017 (97.86%)	14,790 (98.13%)	17,807	
Yes, No. (%)	66 (2.14%)	282 (1.87%)	348	
Lung Cancer				0.03
No, No. (%)	3079 (99.87%)	15,009 (99.58%)	18,088	
Yes, No. (%)	4 (0.13%)	63 (0.42%)	67	
Smoking Status				< 0.01
No, No. (%)	2633 (85.4%)	13,877 (92.07%)	16,510	
Yes, No. (%)	450 (14.6%)	1195 (7.93%)	1645	
Influenza Vaccination				< 0.01
No, No. (%)	3021 (97.99%)	14,266 (94.65%)	17,287	
Yes, No. (%)	62 (2.01%)	806 (5.35%)	868	
Pneumonia Vaccination				< 0.01
No, No. (%)	2892 (93.80%)	11,729 (77.82%)	14,621	
Yes, No. (%)	191 (6.20%)	3343 (22.18%)	3534	

Chi-Square and Fisher's Exact Test are both used to analyze relationships between categorical variables.

**Fig. 1:**
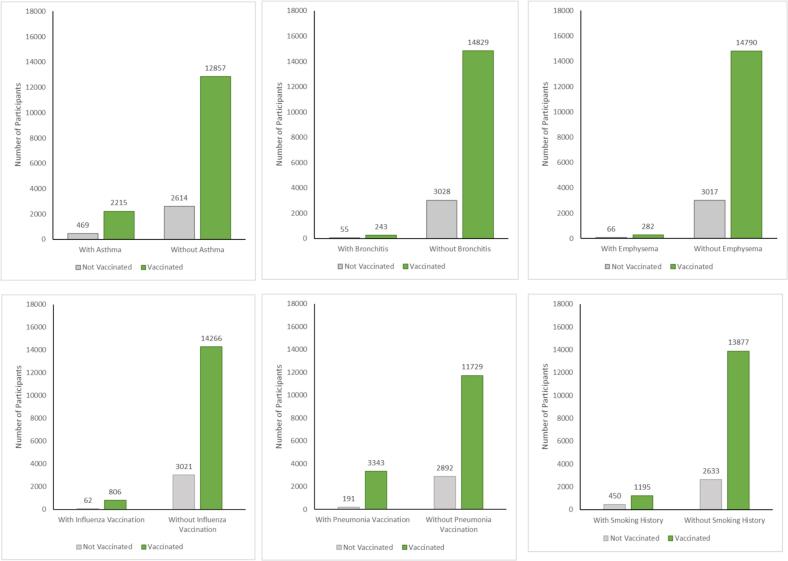
COVID-19 Vaccination Status Among US Adults by Respiratory Disease, Vaccination History, and Smoking Status (Medical Expenditure Panel Survey, 2022).

Also, compared to the vaccinated group, unvaccinated individuals were more likely to be male, non-Hispanic Black, non-immigrants, have lower income, be less educated, reside in the South, and be smokers. Unvaccinated individuals were less likely to receive influenza vaccinations (97.99% not vaccinated vs. 94.65%) or pneumonia vaccinations (93.80% vs. 77.82%). In contrast, the prevalence of respiratory diseases (asthma, bronchitis, emphysema) and asthma alone did not significantly differ between the two groups (*P* > 0.05). However, a statistically significant difference was noted in the prevalence of lung cancer (*P* = 0.03), with a lower proportion of lung cancer reported in the unvaccinated group (0.13%) compared to the vaccinated group (0.42%). Fig. 1 also illustrates COVID-19 vaccination status among all participants by respiratory disease, influenza and pneumonia vaccination, and smoking history.

Primary model validation demonstrated adequate performance with a pseudo-R^2^ of 0.13, AIC of 14,457.76, cross-validation accuracy of 83.00%, and out-of-sample error rate of 16.93% (see Table A3, Appendix). We used unweighted models and included only participants with complete data on vaccination status and key covariates. Some subgroup analyses had limited sample sizes, requiring cautious interpretation of estimates with wider confidence intervals.

Table 2, Table 3 present the results of the primary multivariable logistic regression model predicting COVID-19 vaccination uptake among all participants. Table 2 focuses on respiratory and vaccination history, while Table 3 details socioeconomic factors. The results showed that influenza vaccination (OR = 1.82, 95% CI [1.39, 2.42]), pneumonia vaccination (OR = 2.55, 95% CI [2.14, 3.04]), were significantly associated with higher COVID-19 vaccination uptake. Smoking was associated with lower COVID-19 vaccination uptake (OR = 0.66, 95% CI [0.58, 0.76]). Respiratory diseases (asthma, bronchitis, and emphysema) were not significantly associated with COVID-19 vaccination uptake.

**Table 2:** Multivariable logistic regression: association of respiratory disease and prior vaccination with COVID-19 vaccine uptake among US adults (Medical Expenditure Panel Survey, 2022).

Variables	Odds Ratio	95% Confidence Interval
Prior Vaccination History		
Influenza Vaccination	1.82	[1.39, 2.42]
Pneumonia Vaccination	2.55	[2.14, 3.04]
Respiratory Diseases		
Asthma	1.06	[0.94, 1.19]
Bronchitis	0.93	[0.67, 1.30]
Emphysema	0.78	[0.58, 1.07]
Lung Cancer	2.41	[0.95, 8.11]

Note: Model adjusted for age, sex, race/ethnicity, immigration status, income level, education level, geographic region, and smoking status.

**Table 3:** Multivariable logistic regression: association of socioeconomic and demographic factors with COVID-19 vaccine uptake among US adults (Medical Expenditure Panel Survey, 2022).

Variables	Odds Ratio	95% Confidence Interval
*Smoking Status*		
Smoking History	0.66	[0.58, 0.76]
Age Group (Ref: 17–25)		
26–34	0.64	[0.55, 0.74]
35–54	0.84	[0.73, 0.96]
55–64	1.35	[1.15, 1.57]
> 65	2.19	[1.87, 2.57]
Sex		
Female	1.14	[1.05, 1.24]
Race/Ethnicity (Ref: White)		
Black	1.41	[1.25, 1.60]
Hispanic	1.58	[1.39, 1.80]
Other	1.49	[1.25, 1.78]
Immigrant Status		
Immigrant (Foreign-born)	1.68	[1.47, 1.93]
Income Level (Ref: Lower class)		
Lower Middle Class	1.35	[1.22, 1.50]
Middle Class	1.63	[1.41, 1.89]
Upper Middle Class	2.17	[1.78, 2.67]
Upper Class	2.54	[1.83, 3.61]
Education Level (Ref: No degree)		
Advanced Degrees	7.11	[4.41, 12.24]
Bachelor's Degrees	4.30	[3.67, 5.05]
Grade School	1.07	[0.88, 1.32]
High School	1.35	[1.20, 1.51]
Some Colleges	1.71	[1.45, 2.02]
Region (Ref: Midwest)		
Northeast	1.68	[1.44, 1.95]
South	0.91	[0.82, 1.02]
West	1.30	[1.15, 1.48]

Note: These Odds Ratios (ORs) are derived from the same multivariable logistic regression model as Table 2. Estimates for socioeconomic variables are adjusted for one another and for respiratory health factors (asthma, bronchitis, emphysema, lung cancer, and prior vaccination). These should be interpreted as direct associations conditional on the other covariates in the model, rather than total causal effects.

Table 4 presents the results of the logistic regression model predicting COVID-19 vaccination uptake among participants with and without respiratory disease. The model showed that among participants with at least one respiratory disease, prior influenza vaccination (OR = 1.78, 95% CI [1.05, 3.21]) and pneumonia vaccination (OR = 2.09, 95% CI [1.50, 2.94]), were significantly associated with higher COVID-19 vaccination uptake. Smoking was associated with lower COVID-19 vaccination uptake (OR = 0.53, 95% CI [0.40, 0.70]), while lung cancer was not significantly associated with COVID-19 vaccination uptake. Also, among participants without respiratory disease, influenza vaccination (OR = 1.87, 95% CI [1.37, 2.61]), and pneumonia vaccination (OR = 2.79, 95% CI [2.28, 3.44]), were significantly associated with higher COVID-19 vaccination uptake. Conversely, Smoking was associated with lower COVID-19 vaccination uptake (OR = 0.70, 95% CI [0.61, 0.82]). Lung cancer was not significantly associated with COVID-19 vaccination uptake. Table 5 presents the results of the logistic regression model for COVID-19 vaccination uptake among participants with and without prior influenza vaccine history. The model revealed that among participants with influenza vaccine history, prior pneumonia vaccination (OR = 4.79, 95% CI [2.51, 9.37]) was significantly associated with higher COVID-19 vaccination uptake. Also, among participants without influenza vaccine history, prior pneumonia vaccination (OR = 2.51, 95% CI [2.10, 3.01]) was significantly associated with higher and smoking (OR = 0.65, 95% CI [0.57, 0.64]) was significantly associated with lower COVID-19 vaccination uptake.

**Table 4:** Multivariable logistic regression: association of respiratory disease and prior vaccination with COVID-19 vaccine uptake among US adults by respiratory disease (Medical Expenditure Panel Survey, 2022).

Variables	At Least One Respiratory Disease		Without any Respiratory Disease	
	Odds Ratio	95% Confidence Interval	Odds Ratio	95% Confidence Interval
Influenza Vaccination				
Yes	1.78	[1.05, 3.21]	1.87	[1.37, 2.61]
Pneumonia Vaccination				
Yes	2.09	[1.50, 2.94]	2.79	[2.28, 3.44]
Lung Cancer				
Yes	2.96	[0.84, 18.82]	2.16	[0.61, 13.83]
Smoking Status				
Smoking History	0.53	[0.40, 0.70]	0.70	[0.61, 0.82]

Note: Models are adjusted for age, sex, race/ethnicity, immigration status, income level, education level, and geographic region.

**Table 5:** Multivariable logistic regression: association of respiratory disease and prior vaccination with COVID-19 vaccine uptake among US adults by influenza vaccine history (Medical Expenditure Panel Survey, 2022).

Variables	With Influenza Vaccine History		Without Influenza Vaccine History	
	Odds Ratio	95% Confidence Interval	Odds Ratio	95% Confidence Interval
Pneumonia Vaccination				
Yes	4.79	[2.51, 9.37]	2.51	[2.10, 3.01]
Respiratory Diseases				
Asthma	0.73	[0.36, 1.56]	1.07	[0.95, 1.21]
Bronchitis	0.43	[0.13, 1.54]	0.98	[0.70, 1.40]
Emphysema	0.78	[0.25, 2.89]	0.78	[0.57, 1.07]
Lung Cancer	NA*		2.30	[0.90, 7.78]
Smoking Status				
Smoking History	1.16	[0.56, 2.57]	0.65	[0.57, 0.74]

Note: * Estimates for Lung Cancer in the “With Influenza Vaccine History” subgroup were inflated and unstable due to small sample size; therefore, reliable Odds Ratios could not be calculated (NA). Models are adjusted for age, sex, race/ethnicity, immigration status, income level, education level, and geographic region.

## 4 Discussion

This nationally representative analysis of US adults shows that prior vaccination history, particularly influenza and pneumonia vaccination, was significantly associated with increased COVID-19 vaccination uptake, while respiratory disease presence was not. Socioeconomic factors such as higher income and education were strongly associated with vaccination acceptance across all subgroups. These findings highlight the importance of routine immunization programs as potential pathways to broader vaccine coverage and suggest that vaccination behavior may be more likely influenced by established health-seeking patterns than by specific disease risk factors.

Our findings are consistent with prior research investigating the effect of socioeconomic factors' effect on COVID-19 vaccination uptake (Aw et al., 2021; Soares et al., 2021). A scoping review evaluated COVID-19 vaccine hesitancy and its determinants among high-income countries and reported that younger age, being female, not being of white ethnicity, and lower education were associated with increased vaccine hesitancy (Aw et al., 2021). In addition, several factors, including being younger, loss of income during the pandemic, and having no intention of taking the influenza vaccine, were associated with both refusal and delay in COVID-19 vaccination in Portugal (Soares et al., 2021). Another study among Medicare enrollees across counties in the US found that Black/African Americans had the lowest influenza immunization rates, and also, social vulnerability (e.g., lower income, lower education levels) decreases rates of influenza vaccination in minority communities (Tatar et al., 2023).

While patients with prior respiratory diseases are at higher risk of developing severe COVID-19 and negative health outcomes compared to the general population (Beltramo et al., 2021; Bouloukaki et al., 2024; Liang and Sun, 2023), the association between prior respiratory diseases and vaccine uptake appears to be mixed. We did not find a significant association between respiratory diseases and COVID-19 vaccination uptake; however, some studies reported that patients with asthma who had worse clinical status, including respiratory symptoms (e.g., cough, breathlessness, and chest tightness) and functional status (e.g., activity, sleep, and energy levels), and also patients with poorly controlled asthma, and individuals using biologic therapies were more likely to be hesitant about getting vaccinated (Bouloukaki et al., 2024; Chang et al., 2023). While immunological diseases are not a contraindication for COVID-19 vaccination, there are situations involving severe allergic reactions or acute immunological distress (e.g., acute severe illness) where physicians may suggest avoiding, delaying, or monitoring more strictly after COVID-19 vaccination (Bian et al., 2022; Yan et al., 2021). One study in China reported that the COVID-19 vaccination rate in asthmatic patients was lower than that of the general population (Chang et al., 2023). Our findings are consistent with another study in Greece that found that individuals with asthma or Chronic Obstructive Pulmonary Disease (COPD) who had previously received the influenza vaccine or more than three COVID-19 vaccine doses were positively associated with regular COVID-19 vaccinations (Beltramo et al., 2021). In addition, our results showed that individuals with a history of smoking had lower COVID-19 vaccination uptake among all participants and among participants with at least one respiratory disease. Individuals with a history of smoking are less likely to adhere to preventive measures in general and have lower adherence to other vaccines (Peña et al., 2024). Despite smokers having higher risks of COVID-19 hospitalizations and deaths (Neira et al., 2021; Patanavanich et al., 2023), the use of cigarettes, e-cigarettes, and marijuana has not been reported to be associated with COVID-19 hesitancy (Yang et al., 2021).

Despite the assumption that individuals with severe disease and underlying comorbidities (e.g., cancer, autoimmune diseases, and chronic lung diseases) would be more likely to accept the COVID-19 vaccination, these individuals remained hesitant about the COVID-19 vaccine (Tsai et al., 2022). Similarly, we did not find a significant association between lung cancer and COVID-19 vaccination uptake. COVID-19 vaccination hesitancy rates in cancer patients vary significantly cross-culturally, and cancer patients have unique concerns about vaccination, including interactions with cancer treatments (Tsai et al., 2022). Misconceptions in patients with lung cancer contribute to COVID-19 vaccine hesitancy (Zhuang et al., 2021). Even with demonstrated data on the efficacy of COVID-19 vaccines at preventing COVID-19 illness, including severe disease, and minimal safety concerns (Baden et al., 2021), some sociodemographic groups are differentially impacted by exposure to misinformation, and scientific-sounding misinformation is more strongly associated with declines in vaccination intent (Loomba et al., 2021).

This study utilizes the strength of the nationally representative MEPS dataset to better understand COVID-19 vaccination uptake in the US adult population. By incorporating socioeconomic, respiratory, and vaccination history factors, we aimed to characterize the determinants involved in COVID-19 vaccine hesitancy. Given its cross-sectional design, this study is limited to identifying associations rather than establishing causal relationships. The reliance on self-reported data, particularly self-reported vaccination status, which was not validated against medical records, introduces the potential for reporting and measurement bias. Also, the MEPS dataset does not allow us to determine the temporal order of respiratory illness diagnosis and COVID-19 vaccine uptake. Accordingly, study findings should be interpreted with caution, and we acknowledge this as a limitation of our analysis. As with all observational studies, the influence of unmeasured confounders cannot be entirely ruled out. Furthermore, some subgroup analyses had limited sample sizes, requiring cautious interpretation of estimates with wide confidence intervals. We also acknowledge that sparse data in certain subgroups may affect the precision of some estimates, particularly odds ratios. Because the MEPS focuses on the non-institutionalized population and excludes specific groups (e.g., military personnel and institutionalized populations), the findings have limited generalizability to the entire U.S. population. Its overlapping longitudinal design may also affect sample size and should be considered when interpreting results or guiding policy.

The results of this study suggest that promoting routine vaccinations, particularly influenza vaccination, may serve as a pathway to improving COVID-19 vaccine coverage. Our findings indicate that targeted outreach to individuals with lower socioeconomic status may be a more effective approach for increasing vaccine uptake than disease-specific campaigns for respiratory conditions.

## 5 Conclusions

Prior vaccination history appears to be a stronger predictor of COVID-19 vaccine uptake than the presence of respiratory diseases among US adults. Individuals with a history of influenza and pneumonia vaccinations were significantly more likely to accept the COVID-19 vaccine, while the presence of underlying respiratory diseases did not significantly affect COVID-19 vaccine uptake. These findings highlight the importance of routine immunization programs as a strategy for broader vaccine coverage. Finally, it appears that addressing socioeconomic disparities through targeted interventions may be a more effective strategy for improving vaccine coverage than disease-specific approaches.

## Data sharing statement

No additional data are available.

## Patient and public involvement statement

No patients and the public were involved in this research, and the article does not involve human participants and does not contain personal medical information.

## CRediT authorship contribution statement

**Moosa Tatar:** Writing – review & editing, Writing – original draft, Visualization, Validation, Supervision, Project administration, Methodology, Investigation, Data curation, Conceptualization. **Shahryar Karimi:** Writing – review & editing, Validation, Software, Methodology, Investigation, Formal analysis, Data curation, Conceptualization. **Masoud Valizadeh:** Writing – review & editing, Validation, Methodology, Investigation, Formal analysis, Data curation, Conceptualization. **Abhishek Deshpande:** Writing – review & editing, Validation, Methodology, Investigation, Formal analysis.

## Disclaimer

The views or opinions expressed herein are those of the author and shall not be attributed to and/or does not imply endorsement, recommendation or approval by the Options Clearing Corporation - a systemically important financial market utility.

## Funding sources

The authors have no financial disclosures to report.

## Declaration of competing interest

The authors declare that they have no known competing financial interests or personal relationships that could have appeared to influence the work reported in this paper.

## Figures and Tables

**Table A1:** Definition of respiratory diseases and vaccinations in US adults (Medical Expenditure Panel Survey, 2022).

Diagnosis	Description	Source	ICD-10 CODES
Asthma	Asthma diagnosis	PE30L	J45
Bronchitis	indicating whether a person (aged 18 or older) has had chronic bronchitis in the last 12 months	PE330	J20
Emphysema	Emphysema diagnosis	PE30G	J43
Lung Cancer	Cancer diagnosed - lung	PE140	C34
Influenza Vaccination	Never had Influenza vaccine	DCS Q6	NA
Pneumonia Vaccination	Ever had Pneumonia vaccine	SAQ M-Q44⁎ SAQ F-Q48⁎⁎	NA
COVID-19 Vaccination	Ever had COVID-19 vaccine or booster shot	CV70–80, AH91–93⁎⁎⁎, CV90	NA

⁎ Self-Administered Questionnaire (SAQ).

⁎⁎ Diabetes Care Survey (DCS).

⁎⁎⁎ Additional Healthcare Questions (AH).

**Table A2:** Definition of variables in US adults (Medical Expenditure Panel Survey, 2022).

Variable	Definition
Covid Vaccine Uptake	Indicating whether the individual has received the vaccine
Age	Age of the individual at the time of the survey (minimum eligible age: 17, maximum age: 85)
Age Group	Individuals are categorized in the following age groups: 17–25 years, 26–34 years, 35–54 years, 55–64 years, ≥ 65 years
Sex	Sex assigned at birth (Male, Female)
Race/Ethnicity	White, non-Hispanic – Black, non-Hispanic - Hispanic or Latino - Other/Multi-race (includes Asian, other single or multiple other races)
Minority Status	Indicates minority status
Immigrant	Individuals not born in the USA (foreign-born)
Income	Level of income; Lower class: less than or equal to $30,000, Lower middle class: $30,001 – $58,020, Middle class: $58,021 – $94,000, Upper middle class: $94,001 – $153,000, Upper class: greater than $153,000
Education Level	Level of education; No degree, Grade school, High school, Some college, Bachelor's degree, Advanced degrees
Years of Education	Total number of years of formal education received by the individual
Region	Census region: Midwest, Northeast, South, West
Asthma	Individuals with asthma history
Bronchitis	Individuals with bronchitis history
Emphysema	Individuals with emphysema history
Lung Cancer	Individuals with lung cancer history
Smoking Status	Individuals with smoking history
Influenza Vaccination	Individuals with Influenza vaccination history
Pneumonia Vaccination	Individuals with pneumonia vaccination history

**Table A3:** Goodness of fit and prediction evaluation in US adults (Medical Expenditure Panel Survey, 2022).

Measure	First model (All participants)	Second model (participants with respiratory disease)	Third model (participants with prior influenza vaccination)
Null Deviance	16,542.60	2755.70	446.71
Residual Deviance	14,399.76	2355.72	369.68
Pseudo R-squared	0.13	0.15	0.17
Akaike Information Criterion	14,457.76	2407.72	421.68
Bayesian Information Criterion	14,684.15	2563.56	545.60
Cross-Validation Accuracy	83.00%	83.14%	92.69%
Out-of-Sample Error	16.93%	16.98%	7.31%

## Data Availability

Data will be made available on request.

## References

[bb0005] Abba-Aji M., Stuckler D., Galea S., McKee M. (2022). Ethnic/racial minorities’ and migrants’ access to COVID-19 vaccines: a systematic review of barriers and facilitators. J. Migration Health.

[bb0010] Aw J., Seng J.J.B., Seah S.S.Y., Low L.L. (2021). COVID-19 vaccine hesitancy—a scoping review of literature in high-income countries. Vaccines.

[bb0015] Baden L.R., El Sahly H.M., Essink B. (2021). Efficacy and safety of the mRNA-1273 SARS-CoV-2 vaccine. N. Engl. J. Med..

[bb0020] Barri S., Al-Dahir S., Heyer K. (2025). Influence of respiratory disease experiences on COVID-19 vaccine acceptance: a study from southeastern Louisiana. Front. Public Health.

[bb0025] Beltramo G., Cottenet J., Mariet A.-S. (2021). Chronic respiratory diseases are predictors of severe outcome in COVID-19 hospitalised patients: a nationwide study. Eur. Respir. J..

[bb0030] Bian S., Li L., Wang Z. (2022). Allergic reactions after the administration of COVID-19 vaccines. Front. Public Health.

[bb0035] Bouloukaki I., Christodoulakis A., Papageorgakopoulou S., Tsiligianni I. (2024). The prevalence and determinants of hesitancy for regular COVID-19 vaccination among primary healthcare patients with asthma or COPD in Greece: a cross-sectional study. Vaccines.

[bb0040] Centers for Disease Control and Prevention. People with Certain Medical Conditions and COVID-19 Risk Factors. COVID-19 2025; https://www.cdc.gov/covid/risk-factors/index.html#:∼:text=Like%20adults%2C%20children%20and%20teens,very%20sick%20from%20COVID%2D19. Accessed July 11, 2015.

[bb0045] Chang C., Zhang X., Feng Y. (2023). COVID-19 vaccine uptake and hesitancy in Chinese patients with asthma. J. Asthma.

[bb0050] Chirico F., da Silva J.A.T., Tsigaris P., Sharun K. (2022). Safety & effectiveness of COVID-19 vaccines: a narrative review. Indian J. Med. Res..

[bb0055] Dudley M.Z., Halsey N.A., Omer S.B. (2020). The state of vaccine safety science: systematic reviews of the evidence. Lancet Infect. Dis..

[bb0060] Islam M.S., Kamal A.-H.M., Kabir A. (2021). COVID-19 vaccine rumors and conspiracy theories: the need for cognitive inoculation against misinformation to improve vaccine adherence. PLoS One.

[bb0065] Khan A, Zhu Y, Babcock HM, et al. COVID-19 and influenza vaccine hesitancy among adults hospitalized in the United States, 2019–2022. Vaccine. 2025:126806. 10.1016/j.vaccine.2025.126806 PMC12229716 39884913

[bb0070] Lazarus J.V., Wyka K., White T.M. (2023). A survey of COVID-19 vaccine acceptance across 23 countries in 2022. Nat. Med..

[bb0075] Liang Y., Sun Y. (2023). Awareness of and attitude toward COVID-19 vaccination among individuals with COPD and the strategies to overcome vaccine hesitation: a mini review. Hum. Vaccin. Immunother..

[bb0080] Loomba S., De Figueiredo A., Piatek S.J., De Graaf K., Larson H.J. (2021). Measuring the impact of COVID-19 vaccine misinformation on vaccination intent in the UK and USA. Nat. Hum. Behav..

[bb0085] (2024). Medical Expenditure Panel Survey (MEPS).

[bb0090] (2025). Medical expenditure panel survey (MEPS). Medical Expenditure Panel Survey. https://meps.ahrq.gov/mepsweb/.

[bb0095] Neira D.P., Watts A., Seashore J., Polychronopoulou E., Kuo Y.-F., Sharma G. (2021). Smoking and risk of COVID-19 hospitalization. Respir. Med..

[bb0100] Newman P.A., Dinh D.A., Nyoni T. (2025). Covid-19 vaccine hesitancy and under-vaccination among marginalized populations in the United States and Canada: a scoping review. J. Racial Ethn. Health Disparities.

[bb0105] Patanavanich R., Siripoon T., Amponnavarat S., Glantz S.A. (2023). Active smokers are at higher risk of COVID-19 death: a systematic review and meta-analysis. Nicotine Tob. Res..

[bb0110] Peña S., Zhou Z., Kestilä L. (2024). Tobacco use and uptake of COVID-19 vaccinations in Finland: a population-based study. Nicotine Tob. Res..

[bb0115] Saiphoklang N., Phadungwatthanachai J. (2022). Factors influencing acceptance of influenza and pneumococcal vaccinations for patients with chronic obstructive pulmonary disease. Hum. Vaccin. Immunother..

[bb0120] Soares P., Rocha J.V., Moniz M. (2021). Factors associated with COVID-19 vaccine hesitancy. Vaccines.

[bb0125] Tang S., Ji L., Bishwajit G., Guo S. (2024). Uptake of COVID-19 and influenza vaccines in relation to preexisting chronic conditions in the European countries. BMC Geriatr..

[bb0130] Tatar M., Farokhi S., Araz O.M., Deshpande A., Wilson F.A. (2023). Association of social vulnerability and influenza vaccination rates for annual Medicare enrollees at the county-level in the United States. Prev. Med..

[bb0135] Tavakol M., Gharagozlou S., Abbasi M., Zamani Z., Gharagozlou M. (2024). Pediatric asthma and COVID-19 vaccination: unveiling patterns of hesitancy and acceptance. Ther. Adv. Vaccines Immunother..

[bb0140] Troiano G., Nardi A. (2021). Vaccine hesitancy in the era of COVID-19. Public Health.

[bb0145] Tsai R., Hervey J., Hoffman K. (2022). COVID-19 vaccine hesitancy and acceptance among individuals with cancer, autoimmune diseases, or other serious comorbid conditions: cross-sectional, internet-based survey. JMIR Public Health Surveill..

[bb0150] Yan Z., Yang M., Lai C.-L. (2021). COVID-19 vaccinations: a comprehensive review of their safety and efficacy in special populations. Vaccines.

[bb0155] Yang Y., Dobalian A., Ward K.D. (2021). COVID-19 vaccine hesitancy and its determinants among adults with a history of tobacco or marijuana use. J. Community Health.

[bb0160] Zhuang W., Zhang J., Wei P. (2021). Misconception contributed to COVID-19 vaccine hesitancy in patients with lung cancer or ground-glass opacity: a cross-sectional study of 324 Chinese patients. Hum. Vaccin. Immunother..

